# Abstracts &c.

**Published:** 1889-12

**Authors:** 


### ABSTRACTS AND GLEANINGS.

**A Case of Nutmeg Poisoning.**—On August 14, 1889, I was called to see a boy aged three. I found my little patient with abnormal temperature and respiration; pulse regular, but just a little slow; all the muscles completely relaxed. An attempt to arouse him failed, his head falling in whichever way the body inclined. An examination of the pupils revealed complete dilatation. I felt certain that this condition was the effects of something the child had eaten, and, upon inquiry, was informed that he had, unknown to his mother, appropriated from her spice-box five large nutmegs, which he informed his little sister was his tobacco, and which by noon he had managed to consume, presumably spitting out the bulk of the spice. About 2 P. M. he complained of feeling dizzy and soon fell asleep, and they had been unable to arouse him. He had had one movement from the bowels, and had urinated twice while in this condition. There had been no delirium. He recovered consciousness as if awakening from a natural slumber, but with the greatest dislike for nutmegs, after having slept thirty consecutive hours.—Dr. A. Saywer, in *N. Y. Med. Record.*

**Treatment of Warts.**—Altschul recommends the treatment of warts by Unna's method, namely, mercurial ointment, containing five to ten per cent arsenic. The ointment is spread on linen, and applied over the wart, without leaving any mark. The method is painless. The treatment of warts by arsenical paste is by no means a new one, and its efficacy has been frequently confirmed.—*British Medical Journal.*

**Treatment of Chronic Ulcers of the Leg.**—In the treatment of a chronic ulcer, free the bound down edges, paint the surrounding tissue with equal parts of alcohol and iodine, touch the surface thoroughly with solid nitrate of silver, put the patient to bed, and wrap the limb up in a solution of lead water and laudanum.—Prof. Goss in *College and Clinical Record*, Aug., 1889.

**Exalgine.**—This substance, by Dujardin-Baumetz and Bardel as orthomethylanilide, occurs in fine white needles, or in large plates which are sparingly soluble in cold water, more soluble in hot water, and in diluted alcohol. It is antiseptic and antipyretic. The dose is 4 grains, one to three times daily, dissolved in rum or cognac.—*Duetsche Medicnische Wochenschrift.*

**Styrone as an Antiseptic.**—Dr. H. H. A. Beach has employed this substance, which is a derivative of balsam of Peru, with excellent results as an antiseptic during a period of eleven years. It possesses three important advantages, being efficient, non-poisonous and agreeable in odor. It has been given to dogs in doses of a fluid ounce with no unfavorable result. As a deodorizer of offensive wounds or ulcers, particularly those associated with malignant disease or necrosed bone, it is as prompt, efficient and lasting as either of the poisonous or offensive smelling applications in common use. In ulcerating cancerous growths it may be conveniently sprayed upon the parts too sensitive to bear the douching necessary for clearing the surface of decomposing discharges. The following formula has been found useful in such cases, with the addition of morphine when required:

| | | |
|---|---|---|
| ℞ | Styrone | ʒ i. |
| | Glycerine | ℥ i. |
| | Diluted water | ℥ i. |

To raw surfaces, pure styrone is somewhat irritating; but in the form of an emulsion with olive oil, water or liquid vaseline, it may be applied freely to open wounds. In the pleural and peritoneal cavities where the greatest opportunity is offered a poisonous antiseptic for absorption, styrone in solution may be freely used, without danger, (1-200, 1-100, or 1-50 with water). One part to twelve, with water, is sufficiently strong to completely disinfect a foul and ulcerating surface.—*Boston Medical and Surgical Journal*, Aug. 1, 1889.

**Conjugal Diabetes.**—Dr. Debove read a note at the Societe Medicale des Hospitaux on the coexistence of diabetes in the husband and wife. Of fifty cases of diabetes, Dr. Debove observed five which existed simultaneously in the husband and wife. The same coincidence was established by Dr. Lecorche, who thought that this coincidence can only be explained in two ways: by the use of the same defective alimentation, or by the cares of life shared in common by husband and wife. Dr. Debove said that he could not accept this explanation, as women, from the nature of their habits and the exigencies or their sex, do not live exactly as their husbands. Hence it is that gouty couples are rare. He would therefore ask if diabetes were contagious; but for the present we must rest contented to register the fact. If new facts should show that conjugal diabetes is not a simple coincidence, it is only then one could propose a theory, which for the moment would be premature. Five or six other members also cited one or more cases in which they observed the coexistence of diabetes in the husband and wife.—*Lancet.*

**The Blood-Tax of India.**—Recent official publications of the government of India show that last year five-and-twenty thousand human beings were killed by snakes and wild beasts out of a population of about two hundred million. This number is in accord with the past, and may be looked upon as a regular life-charge upon the country. Human life is so cheap in India, however, that perhaps the destruction of property is more felt by the natives. Thus, in 1887, more than sixty thousand head of cattle were destroyed.

The snake is the chief enemy to the human animal, killing annually about twenty thousand people. Of the wild beasts, the tigers and leopards are estimated together to kill about twelve thousand people and fifty thousand cattle, the higher game being especially the food of the bigger cat. Most of the snake-poisoning is the result of an accident. The serpent strikes when trodden on or otherwise interfered with, and is rarely the first aggressor. But one snake—the *Ophiophagus Elaps*, of India—assails and charges man even more savagely and actively than does the tiger himself.

A feeling of blame against the British rulers is the first emotion which such statistics as these give origin to when read; but the problem is so complex and difficult that its present solution seems impossible. The tiger and leopard ought to be exterminated, although some English lovers of sport urge that they should in fact be protected, and that there should be a "close time" when killing of them should be unlawful; but the chief difficulty is with the snake. He permeates the land—denizen not only of forest, jungle and hill-side, he haunts the cities, nestles in the habitations of men and beasts, roams over fields, roads, and gardens—an ever-present spectre of death. Only by a determined, persistent uprising and continuing battle of a whole people could his numbers be distinctly lessened. But in some places the old serpent-worship lingers, and the Hindoo greets the snake rather as a friend, or honors him as a guest, to be reverenced as well as feared.

Sir Joseph Fayrer believes that there ought to be a special governmental department having charge of this matter, although he himself writes, "The people should bestir themselves." The apathy of the Hindoo seems, however, unconquerable. The tiger and leopard eat the Hindoo, his family, his cattle; the bear and wild boar assault him; the wolf, the cowardly hyena, and the mere jackal lie in wait for his children and other feeble folk, while every hour of the day and the night the snake strikes him with speedy and certain death. Yet he bows his head as though helpless and doomed of the gods. The Anglo-Saxon energy may, perhaps, be able to awake him, but we very much doubt it. It is also not possible that the terrible struggle for the mere necessities of life in which the Hindoos pass their existence, has engendered a sort of race instinct that the land is so overcrowded that death of indviduals is better for the mass, and so infanticide is encouraged and the annual tribute to the minotaur of wild beasts paid with indifference if not with willingness.—*Therapeutic Gazette.*

**Whooping Cough Spasm.**—Heiberg was the first to observe that the raising of the upper jaw is the best method of making the larynx admit air, and he recommended a plan for that purpose, which Kappeler had mentioned before him, and which Naegeli has modified and described as follows: Standing in front of the child, the nurse lays firm hold with the index and middle finger of the ascending ramus of the lower jaw in front of the ear, places both thumbs against the chin, and by strong but gentle traction and pressure moves the lower jaw foward and downward. If the mouth is a little open the jaw may be fixed by placing the thumb or index finger along behind the anterior lower incisors and grasping the chin with the rest of the hand, performing traction as above. In all these cases the left hand rests on the forehead of the patient and performs counter-traction. If the nurse is behind the patient, she may place both thumbs close above the angle of the jaw, the index on the zygomatic arch, and the rest of the fingers on the chin, pushing foward and downward. Immediately the upper jaw is raised the child must be told to draw a deep breath. The plan may be adopted even if the fit comes on during sleep, and and Naegeli says that if so the child does not wake.—*Lancet*.

**Cure of Amenorrhœa: Cause or Coincidence?**—On March 2, 1888, I was consulted by a strong, healthy country girl, aged nineteen, who, however, looked considerably older. I was informed that her menses had stopped at the beginning of December, 1887, and from that time she had been continnally laboring under various disorders. She had menstruated for the first time at sixteen years of age; since that time she always enjoyed good health, and worked very hard in the fields. From the age of twelve she had been obliged to work hard to support herself and her mother. The symptoms of which she complained were frequent headache, sometimes with intolerance of light, throbbing in the head, severe pains in the side and back, irregularity of the bowels, derangement of the appetite, and impairment of strength. She informed me that every month a menstrual molimen occurred, consisting in rigors, pains in the back and loins, weight at the lower part of the abdomen, lassitude, and dyspnea, but without any discharge. All these symptoms passed away in two or three days, to be succeeded by frequent headache and depression of strength and spirits. After a careful examination, in order to exclude pregnancy, I used preparations of iron, iodine, strychnine, bromide of potassium in large doses, purgatives, leeches to the breast and to os tincæ, stimulants, and hot vaginal injections. None of these remedies, however, afforded any relief to the patient. On January 20th last she fell down from a terrace and knocked her back very badly against the rocks. I was called to attend her, and besides the pain at the back I found that she had a slight discharge from the vagina. I prescribed a liniment to relieve the pain, and ordered perfect rest to the patient. The day after the girl informed me that the menses had returned copiously during the night. Since then menstruation continues regular, and the patient enjoys very good health.—Dr. Inglot, *British Medical Journal*.

**Death or Abortion.**—Gaillard's *Med. Journal* reports a case in which a woman took a drachm of oil of pennyroyal and half as much fluid extract of ergot, to induce abortion. She recovered under the use of morphine and atropine hypodermically, heat externally and free administration of milk. The fetus was not injured, pregnancy proceeding normally thereafter. It is a pity that women can not be convinced that drugs will not produce abortion, except in exceptional cases, unless given in such large doses that the death of the mother is more likely to result than that of the child.—*Ind. Med. Jour*.

**Powder Stains on the Skin.**—The bluish-black spots produced by gunpowder may be removed by painting with a solution of biniodide of ammonium and distilled water, equal parts; then with dilute hydrochloric acid to reach the tissues deeply affected.—*Revue de Therapeutique.*

**Tongue-tie.**—Dr. Tassius, of Berlin, in a communication to the Memorabilien, makes himself responsible for some novel views on the propriety or otherwise of incising the frænum of the tongue. He says that this little operation has often been performed without considering that other and deeper causes, especially those connected with general development, may be accountable for the faulty development of speech than the mere size of the frænum. He considers that this tiny and admirably constructed organ is a correct regulator of the various movements of the tongue, and that if the same be too hastily interfered with, the more delicate movements may be forever destroyed—a momentous fact for those who are destined to become public singers. He advises his medical brethren, therefore, to proceed very cautiously, and not to operate at too early an age, as much improvement may result from the advancement of general development. Only in those cases in which the frænum is abnormally large and tendinous as to cause a fissure in the tip of the tongue may the incision be made at once, but it should be calculated almost mathematically what proportion the depth of the incision should bear to the failure. The author deprecates, therefore, the use of scissors, as not to be depended on, but advocates the use of a small knife, the incision to be made from below upwards. Where the frænum is of a fleshy character and it is, consequently, certain that the child will never gain distinction in singing or elocution, there is, according to Dr. Tassius, no cause at all for immediate interference, unless the deformity causes a real impediment. It is far better to leave such cases—at any rate for some time—entirely to the *vis medicatrix naturæ.*—*Lancet.*

**Ichthyol in Erysipelas.**—Dr. Brunn (*Therapeutishe Monatshefte*, July 1889) has treated with excellent results a case of facial erysipelas by the method recommended by Unna and Nussbaum. The parts around the erysipelatious area are carefully washed with soap and water, and then the following mixture is thoroughly applied to the seat of the disease:

| | | |
|---|---|---|
| ℞ | Ammon. sulpho-ichthyolate. | |
| | Ætheris | ää 5.00 |
| | Collodii | 10.00 |

A few hours after the application the red, shining and œdematous skin became shrunken and of a yellowish-brown color, the pains disappeared, and the temprature fell to the normal.—*Allgemeine Wiener Mediz. Zeitung.* [P. J. R.]

**Veratrum Viride in Pneumonia.**—G. R. Martine, M. D., of Glens Falls, N. Y., read a paper before the last meeting of the American Medical Association on the use of this remedy. He says: "The whole method can be given in five words, viz.: Hold the pulse below eighty. Keep your fever thermometer in your pocket; it is of little or no use in pneumonia. Death does not result from high temperature in pneumonia, but from high arterial actions, resulting extravasation and consolidation and consequent heart failure. The physician who, dallying whith his fever thermometer, endeavors to cure pneumonia by reduction of temperature, will make about as much headway, and will be about as successful as he would in trying to eat soup with a peg awl. You may ask with what remedies do you hold the pulse at this point."

While not decrying the merits of other heart sedatives, the doctor prefers veratrum viride. He thinks that by keeping cardiac action down to eighty during the entire first stage of the disease, death from heart failure, so common about the seventh day, is impossible.—*Medcal World.*

**Salol and Sulphur.**—Salol, two parts to one of starch, is used by Schwimmer to treat simple venereal ulcers, and also initial lesions. It is not efficient, but is devoid of odor unlike iodoform.

Schwimmer uses only oxynaptholic acid for scabies, ten parts to eighty of green soap or lard. It causes no irritation and cures in three or four days. The ointment is rubbed in twice a day, and a bath taken the fourth day. The acid is white, odorless, bitter, insoluble in water, but soluble in glycerine and fatty oils.

Sherwell, of Brooklyn, treats scabies with dry sulphur powder alone. The dust is rubbed into the affected parts at night, and a teaspoonful is sprinkled in the bed. A bath of soap and water is taken twice a week, with change of clothing. He finds this a mild and certain treatment, well adapted to cases where the entire family suffer. Where there is an old itch, with rhinoceros-like skin, the old plan of soaps, alkalies and scrub-brush, with sulphur ointments, are more direct and preferable. This treatment is original, simple, efficient, convenient and prophylactic. It is a nine days' cure rather than a three days. Sulphur causes no dermatitis or eczema.—*Ind. Med. Jour.*

**Washing out the Abdominal Cavity in Peritonitis.**—In a case recorded in this issue of the Journal the thorough drainage of the abdominal cavity is beautifully exemplified, holding out, in a practical way, encouragement in all cases of abdominal abscess, or general peritonitis. A word of caution, though, is needed. Very few cases could be safely left as long as the one reported by Dr. Bellamy before operative interference is resorted to. It would be rare, indeed, to find another example of a spontaneous pointing for drainage in the perineum, however much such a fortunate symptom might be desired to simplify the ultimate proceedings. One or two cases recently occurring under the eye of the writer show that simply washing out the cavity through a canula, with carbolized water, and leaving the tube in for subsequent washings, is safe practice, and saved the life of a patient in imminent peritonitis.

In another case of perityphlitis, with general peritonitis, and perforation of the bowel, the stitching of the bowel and thorough washing of the cavity discharged a lot of stercoraceous pus, and so far removed the existing evil that the patient in a few hours afterwards had an evacuation of the bowels, the first time in many days, although he succumbed to the disease in less than twenty-four hours. Evidently laparotomy was delayed too long here, although the surgeons with rare good fortune opened the cavity immediately over the perforation.

It is safe practice in surgery to aspirate the abdomen in all cases where the symptoms point to accumulation of pus, the diagnosis completed, a laparotomy by antiseptic rule can be undertaken with as much confidence and promise of success, as formerly it was dangerous and unjustifiable. This branch of surgery deserves to be more generally cultivated, and the steps of such operations are now clearly enough defined to justify any well-read physician in applying them, without necessarily having recourse to a specialist.—*N. C. M. Jour.*

**Goitre Treated with Strophanthus.**—My attention was first attracted to the value of strophanthus in goitre in a most singular manner. Last December, Mrs. R——sent for me to treat her for some heart trouble. She was short of breath, suffered from palpitation, and had a very bad capillary circulation. She informed me that digitalis acted like a poison to her. She also showed me her goitre, an enoromous one, and said that her former physician had given her ergot and digitalis for it, without any effect, save to make her deathly sick. She needed a heart tonic, and I prescribed it for her in big doses (ten drops every four hours). I left her, and saw no more of her for three weeks or more. When she did show up, she was much improved, and the most astonishing part of all was, her neck was decidedly smaller. Her breathing was good, and she felt much better, and she was greatly relieved, but never cured, for the simple reason she would not take it any longer.—S. T. Yount, M. D., in *Medical Waif.*

Eight or ten drops each of tincture of cannabis indica and nux vomica, in an ounce of chloroform water, will often produce a "voracious" appetite.—*Ib.*

**A Novel Tobacco Antidote.**—Habitual tobacco users and whiskey drinkers have been cured by the following plan: Those who smoke their first cigarette, say at seven o'clock in the morning, begin by putting it off just ten minutes past the hour for a few days, then make it fifteen or twenty minutes, and so on, until it will be noon and then night before the first one is smoked. If it is slow it is certainly a sure way of tapering off, if faithfully followed.—*Ib.*

**Biborate of Soda in Epilepsy.**—The objections to the long-continued use of the bromides in epilepsy are well known. Substitutes are eagerly sought after. Of the biborate of soda for this purpose, Dr. J. D. Munson, Medical Superintendent of the Northern Michigan Asylum, in his last report says:

"It has been found quite equal to the bromides in controlling the seizures. In some cases it has been found superior. It is prompt in its action, and does not affect badly the general condition, and is usually well borne after the first few doses.

"Biborate of soda tends to constrict the peripheral bloodvessels in a remarkable manner, and to this action is doubtless due its beneficial action in epileptics with high arterial tension, borax is sometimes harmful, while in those with low arterial tension, but strong heart action, it is most apt to be useful."

In cases with weak heart it acts badly, probably by its vaso-motor action, obstructing still more the outward blood. Among the drawbacks to its use, he gives the following:

"Given regularly in moderate doses it is apt to affect the nutrition of the scalp, the hair becoming rough and brittle, and in one or two cases alopecia has followed. A troublesome psoriasis is occasionally induced, which, according to our experience, has not yielded readily to treatment, nor has the administration of Fowler's solution with it always prevented its appearance. In one case a suppurative inflammation of the middle ear was always induced by the use of the biborate."

He advises that the drug be never given before meals, or for long periods continuously. It is well sometimes to alternate its administration with those of the bromides.—*American Lancet.*

**Sulfonal for Night-Sweats.**—Although the number of remedies recommended for night-sweats is very large, it may not be be amiss to give some information regarding a new cure, sulfonal, the soporific recently so warmly recommended. Battrich's attention was first attracted to the subject by the case of a lady eighty years old, to whom he had administered only one quarter gram as a soporific. The lady had been suffering with night-sweats so profuse that her clothes were changed twice every night. After taking this powder, asked him whether he had mixed anything for those sweats in it. Further experiments showed that in most cases night-sweats could be prevented by one grain of sulfonal. He considered the effect of sulfonal similar to that of atropin, but it is wholly free from unfavorable side-effects. Moreover its effect is lasting, the sweats of the second night being much less profuse without sulfonal.—*Brooklyn Med. Jour.*

The *Pacific Record* recommends a liniment for earache composed of five parts of camphorated chloral, thirty parts of glycerine, and ten parts of the oil of sweet almonds. A piece of cotton is saturated and introduced well into the ear, and it is also rubbed behind the ear. The drain is relieved as if by magic, and if there is inflammation it often subsides quickly.—*Medical World.*

Mention has been made heretofore of a new soporific Somnal, and we find the following interesting information regarding it in the *Chemist and Druggist.* Somnal and chloral. It is prepared from chloral, alcohol and answers to the formula $C_7H_{12}Cl_3O_3N$, thus differing from the chloral-uthrane, hitherto used, by the addition of two atoms of carbon and four atoms of hydrogen. Somnal has a melting-point of 40° C., and boils in vacuo at about 145° C. It is not influenced by the addition of nitrate of silver, nor by the action of acids. It is administered in doses of 30 grains, preferably with liquid extract of liquorice, or with syrup of raspberry, as follows:

| | | |
|---|---|---|
| ℞ | Somnal | ʒ iiss. |
| | Aq. distill., ad. | ℥ iii. |
| | Ext. glycyrrhiz. liq. | ʒ v. |

One tablespoonful at night.

Such a 30-grain dose of somnal is said to create within half an hour of its administration a sound sleep of six to eight hours duration, and without any injurious after effect. It is claimed for somnal that it does not affect the digestion, the breathing, or the temperature of the body, and that it possesses all the advantages of urethane and chloral hydrate, without any of their ill effects. Supplies have reached this country, but so far as we know no trials have yet been undertaken with it.—*Notes on New Remedies.*

In a meeting of the charity-physicians of Berlin, May 2d, a paper "On the Treatment of Night-sweats with Camphoric Acid" was contributed by Dr. Leu, and in this and the subsequent discussion only commendatory expressions were voiced. It was related from experience of the attending members that the new remedy was a very valuable one in phthisis, and had been generally employed with success where other remedies had failed in allaying night-sweats. Stress was laid on the fact that its administration was never accompanied with evil side-effects. Dr. Klemperer, in closing the discussion, stated that he had similarly satisfactory results from the use of acid, agaric., and both remedies were valuable additions to therapeutic agents in phthisis.—*Ib.*

**Acute Intussusception in a Child Cured by Operation.**—(Thomas Annandale.) The author records this case in order to emphasize the importance of early operation in cases of acute intestinal obstruction when other means have failed to relieve the condition. In this case enemata and the introduction of a bougie failed to relieve, although very gentle traction from within, after the abdomen had been opened, was sufficient to release the invagination. The author calls attention in this paper to the additonal proof afiorded by this case of the value of traction upon intestines from whithin in certain cases for strangulated hernia, or some other forms of intestinal obstruction. The child, aged three years, had suffered for two days with the following symptoms: Pain in abdomen with vomiting; passage of several ounces of blood per rectum; and upon the left side, toward the lumbar region, an elongated swelling was felt. Examination per rectum discovered a mass on the left side that could be pushed up, but would immediately descend. The abdomen was opened by a central incision about two inches in length. The itussusception was relieved at once by slight traction. Immediately wind was passed freely by the rectum. The child made a perfect recovery.—*Archives Pediatrics.*

**Salol and Bismuth in Diarrhœa.**—With the onset of each season and its accompanying diseases, the practitioner anxiously awaits any remedial agent that will alleviate disease. The present season with its intestinal troubles, deserves our close attention. Lately I have been using salol and bismuth in these cases with excellent results. The first case was one of diarrhœa in an adult, with intense pain and vomiting. Salol, gr. v., bismuth subnitrate, gr. x, was ordered every two hours. After the first powder was taken, there was no vomiting, and after the second no pain. The powders were then taken every four hours, resulting in a speedy cure.

Six cases in adults have been treated successfully in this manner.

In cholera infantum, salol and bismuth has been used in four cases, with one death. This child, aged five months, was brought to me on the fourth day of illness, with a history of from fifteen to twenty passages daily, and almost incessant vomiting. I had very little hope for the child, yet the vomiting was relieved to a great extent. Brain complications soon appeared and the child died.

In the other forms of diarrhœa in children, about twenty cases have been treated with salol and bismuth, with good results. The vomiting when present was kept under control, the number of passages quickly diminished, with relief from pain, and loss of odor of the discharges. In some cases the addition of pulv. ipecac et opii was called for, in others pepsinum purum (P. D. & Co's); again, ext. ergot fld. was given, but in the larger number of cases, salol and bismuth alone were given.

The quantity of salol was, gr. ¼ to gr. ij for a child, gr. iij to gr. vj for an dult; bismuth subnit., gr. j to gr. iv for a child, gr. v to gr. xv for an adult. I have not mentioned bathing, hot and cold applications, and the general treatment pursued in such cases, but briefly report my experience with the above remedies.

In conclusion, I must admit that the results obtained have been much better than under other treatment, although the experience has been with a limited number of cases—Dr. W. J. Cree in *Amer Lancet.*

**Cardiac Displacement.**—"Were you wounded in the Crimea, Pat?"

"No, yer honor; the bullet hit me in the chist and kem out at me back."

"Why, Pat, that would have gone right through your heart."

"Och, faix me heart was in me mouth."

—*American Prac and News.*

**Salix Nigra.**—This remedy has been used with much success as a sexual sedative in the treatment of masturbation, excessive venery, spermatorrhœa, and ovarian disease. As a sexual sedative the fluid extract of the buds is considered the most efficient. Dose, ¼ to 1 fluid dram, not miscible with water. As a general tonic and antiperiodic the fluid extract of the bark is employed with advantage. Parke, Davis & Co. make both these extracts, and will mail to the medical profession, on demand, working bulletin giving botanical description, medicinal activity, use, and notes of cases.—*Prac and News.*

**The new Tenicides.**—Areca nuts are brought from India, Ceylon, and the Philippine Islands. The *Areca catechu*, of which they are the fruit, is a palm. Arecaline is the active principle, and it is to this that the tenicide property of areca nuts is due. In its chemical composition and in its properties this alkaloid bears a marked resemblance to pelletierine, the active principle of pomegranate. Arecaline is an oily volatile liquid, with alkaline reaction, soluble in alcohol, ether, chloroform and water. With acids it forms soluble salts.

Areca nuts are given in the form of powder; the dose is from a dram to a dram and a half. The alkaloid has not yet been administered with tenicide intent.

The rules for the administration of the areca powder are much the same as those given for the administration of pomegranate bark or pelletierine. Arecaline being a poison to the worm, benumbing and paralyzing it for the time, the administration of the nuts must be followed by an active purge to remove the entire worm before it has time to recover from its stupor. It is well to precede the ingestion of the remedy by milk diet, and by a purgative or enema the evening before, that the intestines may be cleared of fecal matters, and that the drug may have a better chance of coming in contact with the worm.

The helminth is generally expelled entire four or five hours after the ingestion of the remedy.

Another tenicide is the fruit of the well-known cocoanut, *Cocos nucifera.* For a long time the albumen of the cocoanut has been prescribed as a tenfuge in countries where the *Cocos nucifera* grows. Berenger-Teraud, however, says that he has made trial of this remedy a great many times, and only once out of twenty-four succeeded in obtaining the expulsion of the head of the worm.

Aariso, of Athens, writes that while he resided in Abyssinia where it is the fashion to have tape-worms, he chanced to discover the tenicide properties of this albumen. On his return to Athens he tested it thoroughly, and always with satisfactory results, the whole worm being expelled dead.—*Boston Med and Surg. Jour.*

*On the excretion into the stomach of Morphine injected subcutaneously.*—(*Berlin Klin, Wochenshrif, '89, 25.*)—Some years ago Hitzig noticed that when a dog vomited in consequence of subcutaneous injections of morphine, and the vomit was licked up by another dog, the latter also vomited soon after. This observation led to an investigation of the subject by his assistant Alt, with results of some practical importance, which are summed as follows: (1.) After the hypodermic injections of morphine the alkaloid is excreted into the stomach. (2.) The excretion can be detected 2½ minutes after the injection; continues plainly for half an hour, then becomes weaker, and ceases altogether after 50 or 60 minutes. (3.) The nausea occurs only after the appearance of the alkaloid in the stomach, and can be avoided by washing out the stomach. (4.) The amount of morphine excreted into the stomach reaches the neighborhood of half the amount injected. (5.) By repeatedly washing out the stomach the constitutional symptoms can be much reduced; in this way dogs can be made to bear doses otherwise certainly fatal. All the preceding has been proved with certainty only on dogs; but on human beings also the author has shown that morphine injected hypodermically appears in the stomach in much the same way, and that washing out the stomach diminishes its effects. It follows from this that in hypodermic morphine poisoning, the stomach should be washed out with the same care as if the dose had been taken by the mouth; also that in medico-legal cases of suspected poisoning by hypodermic injection, the alkaloid must be searched for in the stomach as well as in the blood.—*Omaha Clinic.*

**Acute Articular Rheumatism.**—In the treatment of this disease Dr. H. Linderborn thinks that sodium dithosalicyate No. II is destined to supplant the use of salicylate of soda. "The dithosalicylic acid Nos. I and II are two isomeric bodies, each of which consists of two molecules of sulphur. No. II (sodium salt) is a greyish-white powder, very hygroscopic, and easily soluble without residue in water. According to Huppe a 20 per cent solution kills anthrax bacilli in 45 minutes, in which time the ordinary salicylate has no perceptible effect—similarly with other bacteria. Four cases of polyarticular, and one of the monoarticular rheumatism were treated, also one of gonitis gonorrhica complicated with iridio-chorhoiditis; the dose was 0.2 (3 grains) morning and evening—oftener in the more severe cases. The slighter cases showed disappearance of joint-swelling, pain, and fever in two days, the more severe cases in six days. One case was a relapse after salicylate treatment; nausea and noise in the ears were complained of, severe sweating occurred only when 0.8 gram (12 grs.) were taken *pro die*. The last mentioned of the above cases was from another hospital, and the patient left cured in ten days. The advantages of this drug over salicylate are: stronger action, therefore smaller doses; tolerance by the stomach (the insoluble dithiosalicylic acid is precipitated from sodium salt in an acid solution); and absence of unpleasant after effects.—*Brit. Med. Jour.*

**Perosmic Acid in Epilepsy.**—Dr. Carl Schrœder has administered perosmic acid to eight cases of epilepsy, in doses of one-sixth of a grain daily in pill form, and has published his results in his inaugural thesis presented before the University of Kiel.

His results show that the value of perosmic acid in the treatment of epilepsy is negative. He administered it in eight cases, not one of which can be regarded as cured. Two were somewhat improved, while in the other six no action whatever was perceptible. Dr. Schrœder suggests that, perhaps, if the treatment had been more prolonged the results might have been somewhat more favorable, since his results are by no means as satisfactory as those of others who have experimented with this drug in epilepsy.—*Ther. Gaz.*

**Croup and Diphtheria.**—In regard to the question: Does membranous croup occur apart from diphtheria? Dr. Rands referred to his former publications on this point, and wished merely to repeat that, in all cases of membranous croup he could examine closely, he found invariably that they were somehow or other connected with a case of diphtheria of the fauces, occurring in the neighborhood either before or after. He, therefore, believed that all cases of membranous croup were caused by the diphtheritic virus.—(Dr. Ranke, Munich, *British Medical Journal.*

**Diphtheria.**—*Its Treatment by Menthol.*—Cholewa (*Therap Mon. atshefte*) reports most favorable results from the application of plugs of cotton wet with a twenty per cent oily solution of menthol to the nose in cases of nasal diphtheria. In cases in which syringing of the nose had been impossible on account of its being entirely filled with membrane, this method seemed rapidly to remove the membrane and to bring the diphtheritic process to a standstill.—*American Jonr.*

**Homeriana in Tuberculosis.**—This plant, which belongs to the family of Polygonaceæ, has been employed by Laskoft (*Journal de Medecine de Paris*) with some success in the treatment of tuberculosis. This plant grows wild in Russia, and contains an acrid irritating oil, which appears to be its active principle.

The author has prescribed a decoction or infusion of this plant in affections of the respiratory passages, and especially in bronchitis and tuberculosis. He affirms that of one hundred and twelve cases of tuberculosis he has produced great improvement in ninety, the improvement consisting in the reduction of temperature, diminution of expectoration, while physical examination revealed retrogression of the pulmonary lesions in cases of advanced tuberculosis.

The author claims that this plant will reduce night-sweats, cough, and expectoration. He administers 1 ounce of the infusion or decoction of the homeriana, to be taken diluted in twenty-four hours.

**Epistaxis.**—*Oil of Turpentine as a Syptic in.*—Ernyei (*Pest Med. Chir. Press*) recommends plugging the nose with tampons saturated with oil of turpentine. He employed this procedure with immediate success in three cases which had resisted all the usual remedies. The only drawback of this treatment consists in the turpentine strongly irritating the nasal mucous membrane.—*Med. Chronicle.*

